# Mucosal-Associated Invariant T Cells as a Possible Target to Suppress Secondary Infections at COVID-19

**DOI:** 10.3389/fimmu.2020.01896

**Published:** 2020-08-04

**Authors:** Roman A. Akasov, Evgeny V. Khaydukov

**Affiliations:** ^1^Federal Scientific Research Centre “Crystallography and Photonics” Russian Academy of Sciences, Moscow, Russia; ^2^Laboratory of Biomedical Nanomaterials, National University of Science and Technology “MISIS”, Moscow, Russia; ^3^Center of Biomedical Engineering, Institute of Molecular Medicine, Sechenov University, Moscow, Russia

**Keywords:** COVID-19, SARS-CoV-2, mucosal associated invariant T cells, riboflavin, flavin mononucleotide, FMN riboswitch, immunomodulation, cytokines

## Introduction

Co-infections are of proven importance in the severity of respiratory diseases while their involvement in COVID-19 progression is still little discussed (1). However, one in seven patients with COVID-19 had secondary non-viral infections during hospitalization, and 50% of non-surviving patients had secondary infection in a retrospective cohort study in Wuhan (2). Severe patients with COVID-19 often need invasive mechanical ventilation that takes a long time (on average 9 days), and can lead to infections acquired in the hospital and on the ventilator (1). The role of gut microbiota in the severity of COVID-19 has been also recently discussed, suggesting that a microbial metabolic process in the gut may affect the production of pro-inflammatory cytokines (3). There are some pathways between the microbiota and the host immune system, including TNFα and IFNγ production associated with specific microbial metabolic pathways of palmitoleic acid metabolism and degradation of tryptophan to tryptophol (4), but this area needs further evaluation. Therefore, the study of interactions between the host and microbiota is of great importance for understanding the progression and therapy of COVID-19.

## Mait Cells Activation as a Possible Mechanism Participating in COVID-19 Progression

Mucosal associated invariant T (MAIT) cells are found in the blood, liver, lungs, and mucosa, protecting against microbial activity and infection (5). MAIT cells can be activated via MR1-dependent and MR1-independent pathways. MR1-independent activation requires cytokines (e.g., IL-12 or IL-18) while MR1-dependent activation needs recognition of small molecules of biosynthesis of vitamin B2 (riboflavin) and B9 (folic acid). Activated MAIT cells rapidly produce pro-inflammatory cytokines including IFNγ, TNFα, and IL-17 (6). Typically, MAIT cells are discussed in context of bacterial or fungal infections, as they can induce the immune response when activated with riboflavin precursors in MR1-dependent manner. However, MAIT cells activation was also described for viral infections, including herpes, hepatitis, or lethal influenza (7, 8). In the case of viruses, MAIT activation occurred via MR1-independent pathway as a result of cytokines binding (IL-18 in synergy with IL-12, IL-15, and/or interferon-α/β) (9). No information regarding the increased values of IL-18, IL-12, or IL-15 in patients with COVID-19 was found and, therefore, this possible activation pathway requires further study. On the other hand, the role of MAIT cells resident in lung tissue in children with community-acquired pneumonia was demonstrated (10). Immunity profiling showed that MAIT cells from the bronchoalveolar lavages, but not from the blood, actively produced IL-17. It is important that most patients were diagnosed with adenovirus and *Mycoplasma pneumoniae* while neither adenovirus nor mycoplasma synthesize riboflavin. The authors suggested that MAIT cells were probably activated through commensal microorganisms or co-infecting bacteria in combination with inflammatory cytokines (10). Since only MAIT cells resident in lung tissue but not derived from the blood produced IL-17, the contribution of co-infecting bacteria appears to be more important. It should be noted that targeting IL-17 was recently proposed as a strategy to combat acute respiratory distress syndrome in COVID-19 (11). Therefore, we hypothesize the importance of the study of MAIT cells in blood and especially in lungs, given the evidence that the status of T cells reflects the severity of infection and predict the clinical outcomes in patients with COVID-19 (12, 13). Thus, we suggest that activation of MAIT cells in secondary non-viral infection via the MR1-dependent pathway may be a factor that enhances the progression of COVID-19, and IL-17 production may be one of the mediators.

## Inhibition of Riboflavin Biosynthesis in Microbiota as a Possible Approach to Prevent Mait Activation

Since MR1-dependent activation occurs as a result of recognizing small molecules of riboflavin biosynthesis, inhibition of this pathway appears to be a promising approach to prevent the immune response of MAIT cells. Indeed, an immunomodulatory strategy of herpesviruses that functionally disrupts the immune response was defined for MR1 targeting (14). It was found that riboflavin biosynthesis can be repressed by inhibiting enzymes involved in riboflavin biosynthesis (15) or at the level of transcription through the flavin mononucleotide (FMN) riboswitch (16). The FMN riboswitch is a metabolite-dependent RNA element that directly binds FMN and controls the expression of genes responsible for riboflavin biosynthesis since FMN is riboflavin-5′-phosphate (17).

There are some synthetic or natural compounds that can inhibit FMN riboswitch. Among them, roseoflavin, a pigment originally isolated from Streptomyces davawensis, was discussed as an antimetabolite analog of riboflavin and FMN with antimicrobial properties (16). The other is 5FDQD, a riboswitch-binding analog of flavin that protects mice against *Clostridium difficile* infection without inhibiting healthy bowel flora (18). Double-targeting of the *Staphylococcus aureus* FMN riboswitch with roseoflavin and ribocil-C demonstrated efficacy in a murine model of MRSA (Methicillin Resistant Staphylococcus Aureus) infection (19).

However, FMN or even riboflavin may be also effective in inhibiting riboflavin biosynthesis. A strategy of the oral supplementation with riboflavin may be proposed, given that some bacteria can switch from biosynthesis to uptake of riboflavin when it is environmentally available. It should be noted that riboflavin supplementation (100 mg daily for 3 weeks) in patients with Crohn's disease, a type of inflammatory bowel disease, led to anti-inflammatory effects (20). Activation of innate MAIT cells in inflammatory bowel diseases resulted in a switch in the pattern of cytokine secretion was previously demonstrated (21). Riboflavin supplementation (10 mg/day, p.o) significantly decreased plasma homocysteine, a marker of inflammation and ischemic injury, in the group of elderly people with low riboflavin status (22).

An alternative approach to inhibit overactivation of MAIT cells is a ligand-dependent downregulation of MR1 cell surface expression via retaining MR1 molecules in the endoplasmic reticulum in an immature form (23).

## Discussion

We hypothesize that secondary non-viral infections may enhance the severity of COVID-19 by activating MR1-dependent MAIT cells. MAIT cells promote protection against primary infections through cytokines production (24), as well as mediate protective host responses in sepsis by reducing bacterial burden (25). However, in the case of secondary infections, additive inflammation can exacerbate the situation due to the progression of the cytokine storm. The possible involvement of MAIT cells in the development of undesirable immune response in certain diseases was previously shown (5). Thus, MAIT cells promote inflammation and exacerbate the disease in murine models of arthritis while mice with MR1 deficiency develop a less severe disease compared to control (26). In mice infected with *Helicobacter pylori*, MAIT cells were expanded in the gastric mucosa and adopted the IL-17A- and IFN-γ-producing phenotype, resulting in the gastric progression (27). It is important that MAIT cell activation is detected 2 h after contact with the antigen (28). It should be also noted that MAIT cells are much less frequent in children (<2 y.o.) than in older humans (5). Functional alteration of innate T cells in COVID-19 patients has been described very recently, including a decrease in circulating MAIT cells in blood, which may be a consequence of their recruitment into the airways (29).

If the hypothesis of MR1-dependent MAIT cells activation in secondary infection of COVID-19 is correct, inhibition mechanisms of this activation could be discussed. This can be accomplished by inhibiting the riboflavin biosynthesis in the microbiota (e.g., with roseoflavin or analogs) or by ligand-dependent downregulation of the MR1 cell surface expression in antigen-presenting cells (e.g., with DB28 or analogs (23)). The first option seems easier to implement since the inhibition of riboflavin biosynthesis pathway can lead to toxicity for microbiota, including pathogen bacteria and yeasts, but not to the host.

We must also point out some arguments that contradict our idea. Thus, neutrophils that are recruited early to sites of infections, including COVID-19 infection, can suppress and prevent overactivation of MAIT cells (30). Since repeated MAIT cells stimulation by cytokines (IL-12 and IL-18) was found to enhance IL-17 production by MAIT cells (31), the patients may not benefit from the treatment that suppresses MR1-dependent stimulation. The interactions of MAIT cells with other participants in the immune response are also unclear, as well as the impact of MAIT cells infection at a distant site, i.e., the impact of gut MAIT cells on pulmonary infection or vice versa (32). Therefore, we appeal to the biomedical community to test the hypothesis of MR1-dependent MAIT cells activation as a possible therapeutic approach.

## Author Contributions

Both authors listed have made a substantial, direct and intellectual contribution to the work, and approved it for publication.

## Conflict of Interest

The authors declare that the research was conducted in the absence of any commercial or financial relationships that could be construed as a potential conflict of interest.
